# Biatrial myxoma and multiple organ infarctions combined with Leriche syndrome in a female patient

**DOI:** 10.1186/1471-2261-14-175

**Published:** 2014-12-05

**Authors:** Seung Yeon Min, Young-Hyo Lim, Hyung Tak Lee, Jinho Shin, Kyung-Soo Kim, Hyuck Kim

**Affiliations:** Division of Cardiology, Department of Internal Medicine, College of Medicine, Hanyang University, Seungdong-Gu, Heangdang-Dong 17, 133-070 Seoul, South Korea; Department of Thoracic and Cardiovascular Surgery, College of Medicine, Hanyang University, Seungdong-Gu, Heangdang-Dong 17, 133-070 Seoul, South Korea

**Keywords:** Myxoma, Heart atria, Cerebrovascular accident, Leriche syndrome, Echocardiography

## Abstract

**Background:**

Multiple organ infarctions combined with Leriche syndrome due to embolic particles of myxoma are very rare. There is no definite guideline for immediate medical treatment.

**Case presentation:**

A 36-year-old married female was referred to the emergency department (ED) with severe pain of both lower extremities and gradual decreased mental status. Brain magnetic resonance imaging (MRI) and computed tomography angiography (CTA) revealed acute multiple organ infarctions including the brain, spleen, and bilateral kidneys combined with Leriche syndrome. To evaluate the embolic source, echocardiography was performed and it revealed biatrial myxoma. Because of the risk of progression in systemic embolic events, surgical excision and embolectomy were performed urgently. After the operation, renal function was recovered, and the pain of both limbs was relieved. However, the visual field defect due to the brain infarction remained. She was discharged uneventfully on the fourteenth postoperative day.

**Conclusion:**

This was an extremely rare case of multiple organ infarctions combined with Leriche syndrome as the initial presentation of biatrial myxoma. The treatment of choice for myxoma is surgical excision, but the optimal timing of operations is still controversial in patients who have had recent neurological insults. Echocardiography was useful to clarify the diagnosis and decide on the proper treatment modality: surgical treatment or thrombolysis.

## Background

Biatrial myxoma is found in less than 5% of all myxoma cases, and multicentric biatrial myxoma is especially rare [1]. Furthermore, multiple organ infarctions combined with Leriche syndrome due to embolic particles of myxoma are very rare. Here, we present a patient with biatrial myxoma and multiple organ infarctions combined with Leriche syndrome who underwent immediate surgical treatment with fair functional recovery.

## Case presentation

A 36-year-old married female was brought to the emergency department (ED) after developing severe pain of both lower extremities and gradual decreased mental status during aerobic dance 12 hours prior. She was doing well without any cardiovascular risk factors.

On her arrival at the ED, her blood pressure was 120/80 mmHg and her respiratory rate was 22 breaths/minute. An electrocardiography showed normal sinus rhythm, but a chest x-ray revealed cardiomegaly with enlargement of both atria.

**Figure 1:**
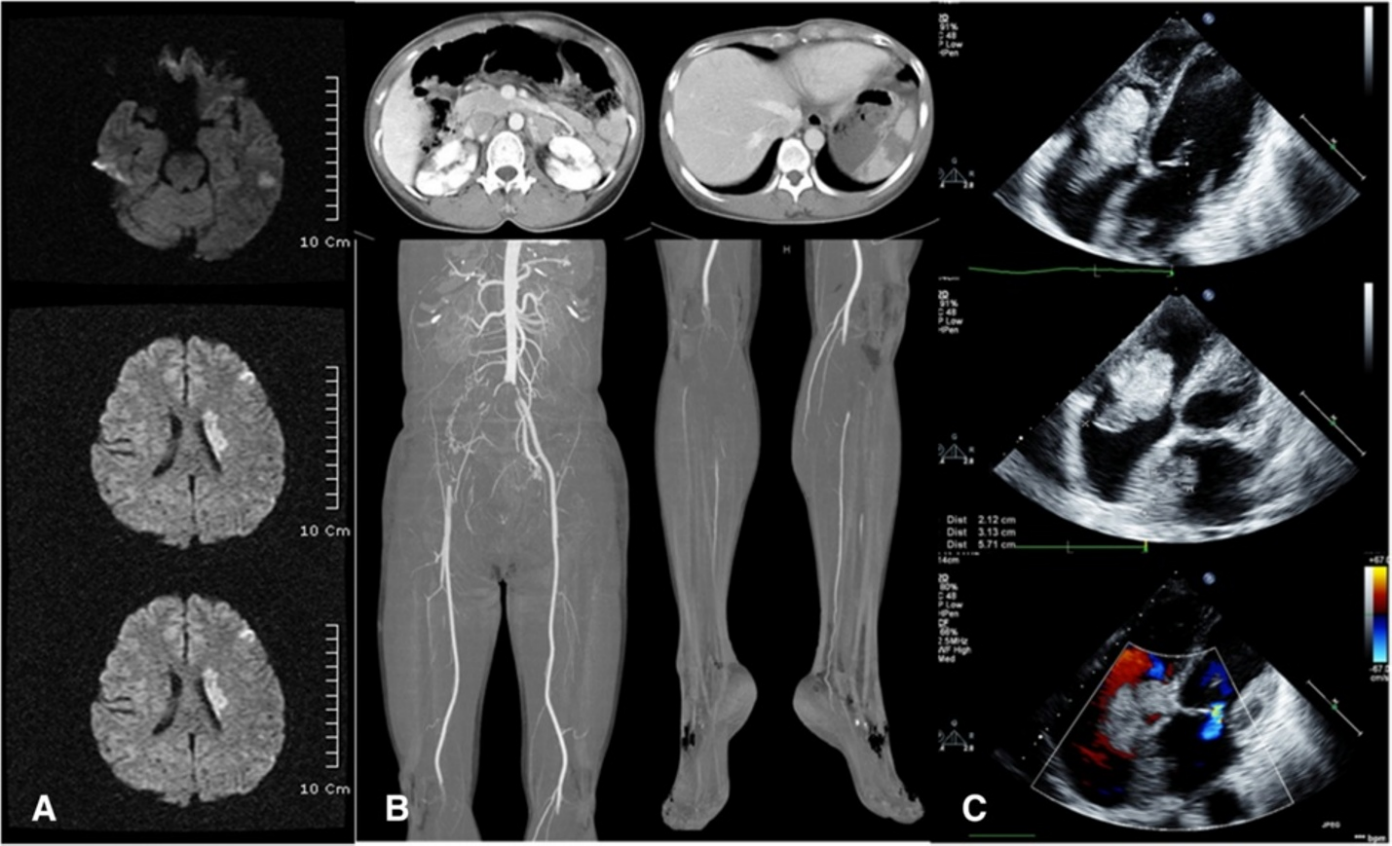
**Brain MRI with diffusion, CTA and transthoracic echocardiography. (A)** Acute multifocal territorial infarct, frontotemporoparietal lobe area and striatocapsule with minimal swelling in the left mid-cerebral artery. **(B)** Multifocal spleen and renal infarctions in bilateral kidneys and complete segmental obstruction of the aortic bifurcation, bilateral common iliac arteries, right internal iliac artery and bilateral popliteal arteries. **(C)** A large (5.7×3 cm) round and pedunculated homogeneous mass that occupied most of the RA and prolapsed through the tricuspid valve with functional tricuspid stenosis, and another large (3.8×2 cm) villous mass that was attached to the septal side with no stalk in the large LA with mild MR.

To evaluate her decreased mental status and pain in both lower extremities, brain magnetic resonance imaging (MRI) with diffusion and computed tomography angiography (CTA) for the lower extremities were conducted. The brain MRI revealed an acute multifocal territorial infarct (Figure 1A). The CTA revealed complete segmental obstruction of the aortic bifurcation, proximal portion of the bilateral common iliac arteries, right internal iliac artery, bilateral popliteal arteries, and multifocal spleen and renal infarctions in the bilateral kidneys (Figure 1B).

To evaluate the multifocal embolic sources, transthoracic echocardiography was conducted. It revealed a large (5.7×3 cm), round, and pedunculated homogeneous mass that occupied most of the right atrium (RA) and prolapsed through the tricuspid valve with functional tricuspid stenosis and another large (3.8×2 cm) villous mass that was attached to the septal side with no stalk in the large left atrium (LA) with mild mitral regurgitation (MR) (Figure 1C). Based on these findings, the patient was diagnosed as having embolic infarctions of multiple organs with huge biatrial myxomas.

**Figure 2:**
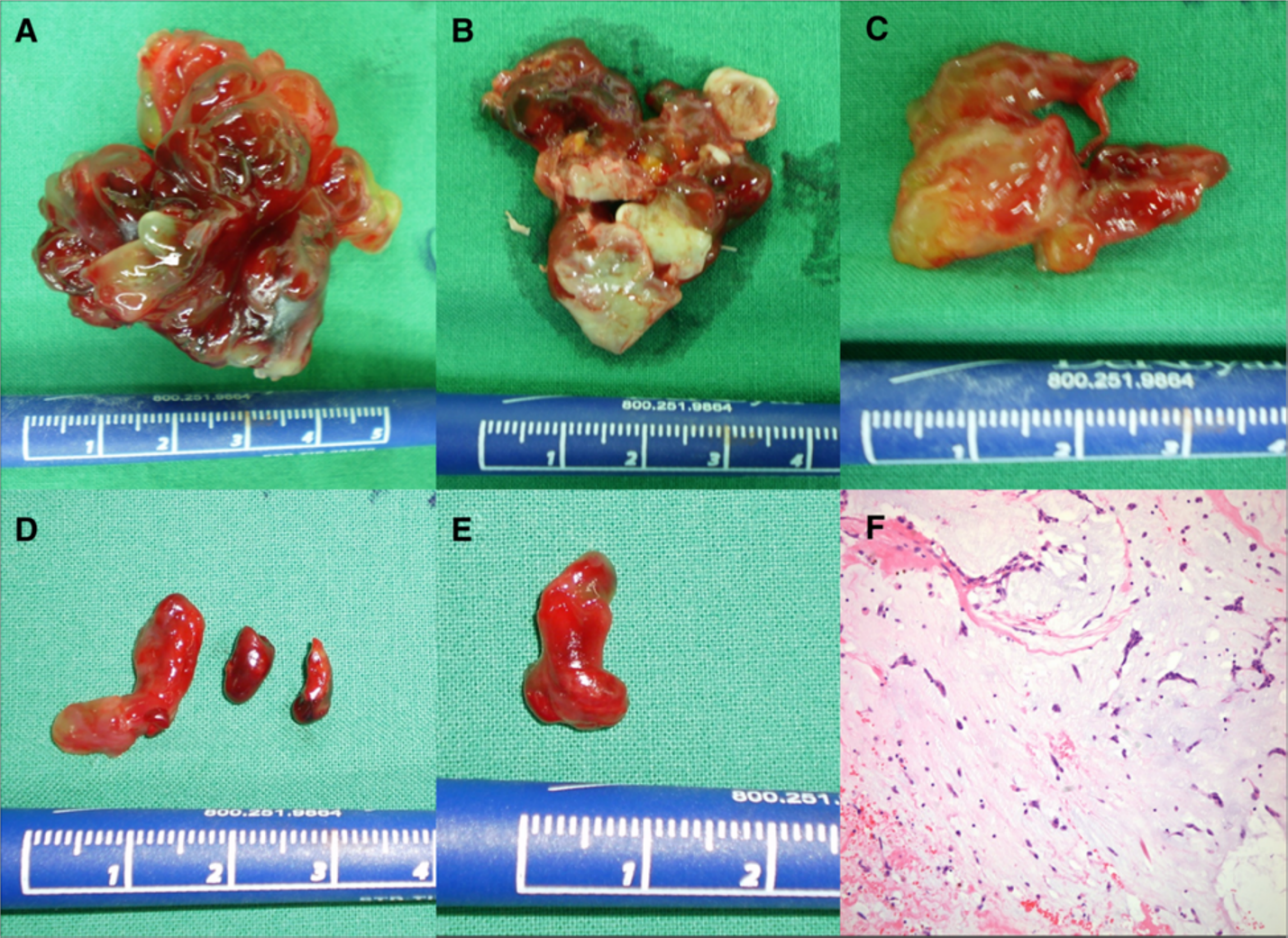
**Surgical (A, B, C, D, E) and histologic (F) findings. (A)** Myxoma from LA, **(B)** Myxoma from RA, **(C)** Embolic myxoma from aortic bifurcation, **(D)** Embolic myxoma from left popliteal artery, **(E)** Embolic myxoma from right popliteal artery. **(F)** Histologic findings of myxoma (H&E x400): Characteristic pale-staining, granular tumor cells containing with an abundant myxoid stroma were noted.

Because of the risk of progression in systemic embolic events, we decided on emergent surgical treatment. At surgery, the biatrial masses were excised, and the tumor emboli in the abdominal aorta, bilateral iliac arteries, femoral arteries, and popliteal arteries were removed using a Fogarty embolectomy catheter. Histologic findings of the biopsy specimen revealed myxoma and tumor emboli from LA myxoma. Surgical excision revealed a 4×4×3 cm RA myxoma with a narrow stalk at the RA posterior wall and a 5×4×3 cm LA myxoma with a broad base attaching to the atrial septum. The histologic findings of myxoma revealed the characteristic finding of pale-staining, granular inflammatory cells (Figure 2). Postoperatively, the patient recovered from the renal infarction and limb ischemia but had a unilateral visual field defect due to the brain infarction. She was discharged uneventfully on the fourteenth postoperative day.

## Discussion

About 75% of myxomas occur in the LA, 15-20% in the RA, 3-4% in the left or right ventricle, and <2.5% in both atria [1, 2]. Polypoid myxomas are usually compact and show little tendency toward spontaneous fragmentation, but they can embolize depending on their mobile state. The less common villous or papillary myxomas have a surface that consists of multiple fine or very fine villous extensions. These extensions are gelatinous and fragile and tend to break off or into pieces, so they are more likely to embolize [1]. As in this case, emboli from the villous LA myxoma with a broad base attached to the atrial septum caused multiple organ infarctions, but the polypoid RA myxoma did not cause embolism.

Cardiac myxoma patients have various features of the triad of constitutional (30%), cardiac (60%), and embolic symptoms (30-40%) [3]. Constitutional symptoms include raised inflammatory markers with fever, weight loss, or symptoms resembling connective tissue disease due to cytokine (interleukin-6) secretion by the myxoma itself, infection, or malignancy [1]. Cardiac symptoms include exertional dyspnea, orthopnea, acute pulmonary edema, syncope, sudden death, and right heart failure. Embolism occurs in 30-40% of patients with myxomas. Since most myxomas are located in the LA, systemic embolism is particularly frequent. In most cases, the cerebral arteries are affected. Occlusions of the peripheral arteries and embolization into visceral, renal, and coronary arteries can also occur [1]. Interestingly, physical exercise can dislodge an embolus from a myxoma of the LA [4].

However, it is rare that multiple organ infarctions combine with Leriche syndrome as the initial manifestation of biatrial myxoma, as in this case. Leriche syndrome is an aortoiliac occlusive disease caused by the occlusion of the abdominal aorta just above the site of its bifurcation [5, 6]. There are two different clinical manifestations of aortoiliac occlusive disease: acute and chronic Leriche syndrome. In acute Leriche syndrome, symptoms usually develop suddenly with symptoms of acute limb ischemia [6].

There are no clear guidelines for immediate medical management. For cases with ischemic stroke and transient ischemic attack, the main issue is early secondary prevention while considering surgery [4]. Anticoagulants and antiplatelet agents are used with the presumption that some of the embolic component is a thrombus but may not be protective [7].

The treatment of choice for cardiac myxoma is surgical excision. Once a diagnosis has been established, surgery should be performed as soon as possible, as the risk of further tumor embolism and valve obstruction is high. The removal of the myxoma in a patient with recent stroke poses a difficult management problem. The concern has been that cardiopulmonary bypass and anticoagulation may exacerbate the neurologic injury. Timing of surgery is still controversial in patients who have had recent neurological insults, and this needs to be clarified as more experience is accrued [4]. In initiating treatment, it was difficult to determine whether the embolic lesion was due to a thrombus or tumor emboli. Emboli from atrial myxoma may be composed of a thrombus, the tumor itself, or both.

However, the echocardiography revealed the answer in this case. In the initial echocardiographic finding, the relatively mild MR and small tumor size compared with the large LA and irregular or more villous tumor margin compared with the right side mass led us to the judgement that the multiple embolic infarction originated from emboli from a huge tumor mass located in the LA. Even if the brain infarction was due to thrombotic embolism, the patient had entered the ED past the golden time of thrombolysis. Therefore, immediate surgical excision of the embolic origin was determined to be the optimal treatment of the condition to prevent further progression of systemic embolism.

## Conclusion

It is rare that multiple organ infarctions combine with Leriche syndrome as the initial manifestation of biatrial myxoma, as in this case. Interestingly, physical exercise can dislodge an embolus from a myxoma of the LA. The treatment of choice for cardiac myxoma is surgical excision. However, the removal of the myxoma in a patient with recent stroke poses a difficult management problem. The concern has been that cardiopulmonary bypass and anticoagulation may exacerbate the neurologic injury. Timing of surgery is still controversial in patients who have had recent neurological insults, and this needs to be clarified as more experience is accrued. Echocardiography was essential in clarifying the diagnosis and determining the proper treatment modality: surgical treatment or thrombolysis.

## Consent

Written informed consent was obtained from the patient to publish this case report and any accompanying images, a copy of which is available for review by the Editor of this journal.

## Abbreviations

ED: Emergency department
MRI: Magnetic resonance imaging
CTA: Computed tomography angiography
LA: Left atrium
RA: Right atrium
MR: Mitral regurgitation.
